# Comparison of trapping profiles between d-peptides and glutathione in the identification of reactive metabolites

**DOI:** 10.1016/j.toxrep.2015.07.002

**Published:** 2015-07-09

**Authors:** Jaana E. Laine, Merja R. Häkkinen, Seppo Auriola, Risto O. Juvonen, Markku Pasanen

**Affiliations:** University of Eastern Finland, Faculty of Health Sciences, School of Pharmacy, Yliopistonranta 1, P.O. Box 1627, FIN-70211 Kuopio, Finland

**Keywords:** Bioactivation, Cytochrome P450, Glutathione, LC/MS liquid chromatography mass spectrometry, Reactive metabolites, Covalent binding, Peptide d-isomer, Peptide adducts

## Abstract

Qualitative trapping profile of reactive metabolites arising from six structurally different compounds was tested with three different d-peptide isomers (Peptide 1, gly–tyr–pro–cys–pro–his-pro; Peptide 2, gly–tyr–pro–ala–pro–his–pro; Peptide 3, gly–tyr–arg–pro–cys–pro–his–lys–pro) and glutathione (GSH) using mouse and human liver microsomes as the biocatalyst. The test compounds were classified either as clinically “safe” (amlodipine, caffeine, ibuprofen), or clinically as “risky” (clozapine, nimesulide, ticlopidine; i.e., associated with severe clinical toxicity outcomes). Our working hypothesis was as follows: could the use of short different amino acid sequence containing d-peptides in adduct detection confer any add-on value to that obtained with GSH? All “risky” agents’ resulted in the formation of several GSH adducts in the incubation mixture and with at least one peptide adduct with both microsomal preparations. Amlodipine did not form any adducts with any of the trapping agents. No GSH and peptide 2 and 3 adducts were found with caffeine, but with peptide 1 one adduct with human liver microsomes was detected. Ibuprofen produced one Peptide 1-adduct with human and mouse liver microsomes but not with GSH. In conclusion, GSH still remains the gold trapping standard for reactive metabolites. However, targeted d-peptides could provide additional information about protein binding potential of electrophilic agents, but their clinical significance needs to be clarified using a wider spectrum of chemicals together with other safety estimates.

## 1 Introduction

The metabolic activation of a drug to an electrophilic reactive metabolite and its covalent binding to cellular macromolecules, such as proteins or nucleic acids, is considered to be one of many ways that drugs exert their toxicity. The adducts formed can cause either acute or long-term toxicity, e.g., degenerative diseases [5], [25], [54]. A thorough toxicological hazard and risk assessment should include determination of the electrophilic potential of a drug and potential for adduct formation, which are related in this type of evaluation. However, it is difficult to monitor the formation of electrophilic reactive metabolites because they are unstable. Furthermore, usually the amounts of these compounds produced are very small compared to major metabolites or the parent molecule. Thus, different types of trapping agents are needed to measure the formation of electrophilic compounds. Glutathione (GSH) is the most commonly used trapping agent [3], [37], [38] because it forms conjugates with many different kinds of electrophiles. Other trapping agents are: *N*-acetylcysteine, [14], [52], potassium cyanide (KCN) [2], [5], [37], [38], semicarbazide [37], [59], methoxylamine [57], *γ*-glutamylcysteinyllysine [56] and some other synthetic peptides [33], [21], [51]. In addition, ferrocenyl-modified glutathione (FP)-GSH has been used instead of GSH because (FP)-GSH has higher retention times than GSH [17]. Furthermore, a bromine-containing glutathione analog [23] has been claimed to increase sensitivity compared to unmodified GSH.

New liquid chromatography–mass spectrometry (LC/MS) techniques are very powerful analytical tools for elucidating the metabolism of xenobiotics [43], [42], [49]. Ion trap instruments with rapid scanning speeds allow the sensitive detection of metabolites and the acquisition of their mass spectra in a single LC/MS run. The triple quadrupole-linear ion trap technique expands the range of metabolite screening [16], [40].

**Fig. 1:**
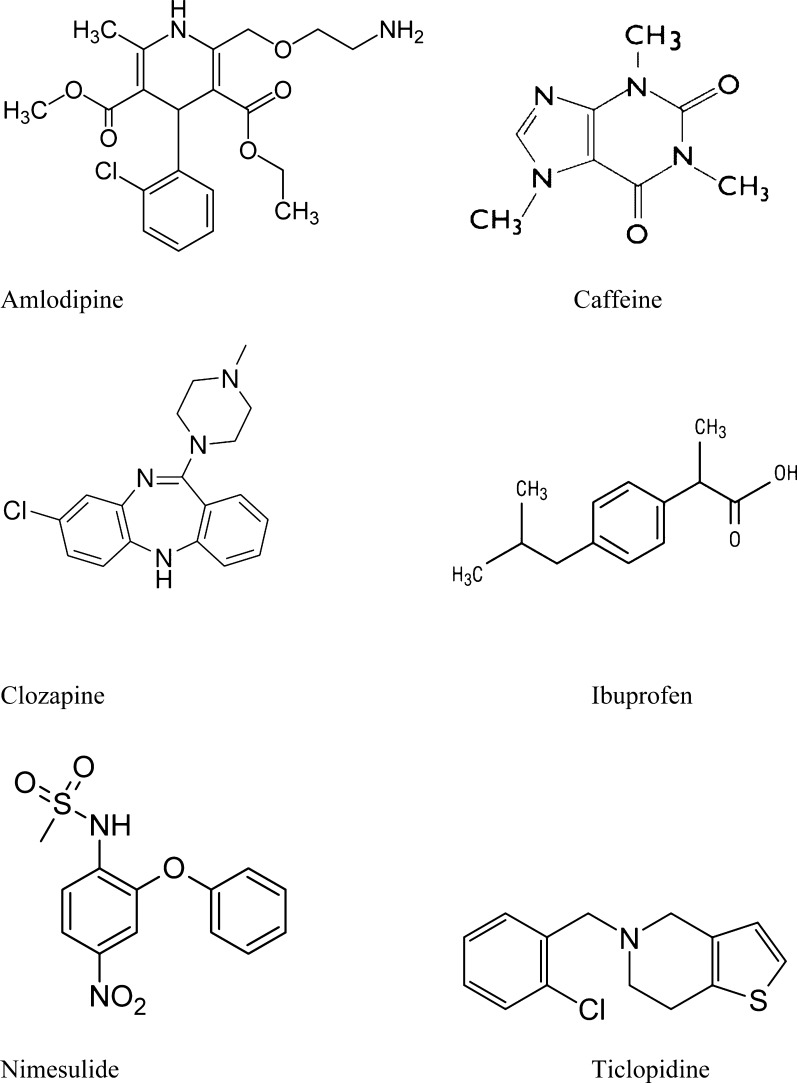
Chemical structures of the test compounds.

In this study, we evaluated the trapping properties of three synthetic d-peptides with different sequences (gly–tyr–pro–cys–pro–his–pro (Peptide 1), gly–tyr–pro–ala–pro–his–pro (Peptide 2) and gly–tyr–arg–pro–cys–pro–his–lys–pro (Peptide 3) and compared them to GSH. Potential electrophiles were produced from three clinically “safe” drugs (amlodipine, caffeine and ibuprofen) and from three clinically “risky” drugs (clozapine, nimesulide and ticlopidine) (Fig. 1) in in vitro incubations using control mouse and human liver microsomes as the enzyme source. The identification of adducts was based on their fragmentation in LC/MS ion-trap mass-spectrometry with an electrospray ionization (ESI) source.

## 2 Materials and methods

### 2.1 Chemicals

Glutathione (GSH), MgCl_2_, NADPH, potassium phosphate, amlodipine, caffeine, clozapine, ibuprofen, nimesulide and ticlopidine were purchased from Sigma–Aldrich chemicals (Helsinki, Finland). Acetonitrile was from J.T. Baker (St. Louis MO, USA) and formic acid from Merck KGaA (Darmstadt, Germany). d-Isomer peptides (gly–tyr–pro–cys–pro–his–pro, gly–tyr–pro–ala–pro–his–pro and gly–tyr–arg–pro–cys–pro–his–lys–pro) were synthesized by GL Biochem (Shanghai, China) and had a purity of >95%.

### 2.2 Biological material

Human liver microsomes were purchased from BD Biosciences (Woburn, MA, USA). DBA/2N/Kuo mice were obtained from the National Laboratory Animal Centre, Kuopio University. Liver microsomes and the cytosolic fraction were prepared from the livers of animals as described previously [22]. The animals had unrestricted access to water and standard chow (Lactamin R36, Lactamin AB, Södertälje, Sweden). The Ethics Committee for Animal Experiments, University of Kuopio approved these experiments.

### 2.3 Adduct formation with six test compounds

The incubation conditions were as described below. Because the enzyme sources used were pooled microsomes of human or mouse origin, the incubations were carried out twice and as duplicates. The 500-μl incubation mixture contained 100 mM potassium phosphate buffer, pH 7.4, 2.5 mM MgCl_2_, 0.5 mM NADPH, 0.30 mg/ml of microsomal protein, the substrate concentration was 50 μM (amlodipine, caffeine, clozapine, ibuprofen, nimesulide and ticlopidine), and the trapping substance concentration was 572 μM for d-peptides 1–3 or 1 mM for GSH. Incubation time was 1 h at 37 °C. The incubation mixture was preheated at 37 °C for 10 min, the oxidation reaction was started by adding NADPH and the reaction was terminated by addition of an equal volume of acetonitrile. Three kinds of controls were used: incubations without NADPH, enzyme source or substrate. The selection of 572 μM peptide trapping concentration was based on the fact that at higher concentrations peptides would cause problems in the LC/MS analysis because the peptide could precipitate in the column and ion source. Unlike GSH, which comes out at very early times in the LC run, the non-adducted peptides cannot be diverted to waste at the beginning of LC separation due to relatively long retention times. The test tubes were centrifuged for 20 min at 2000 × *g*. Supernatant (500 μl) was taken and the acetonitrile was evaporated for 1 h at room temperature in an evaporating centrifuge. The residuals were dissolved in 250 μl of water and centrifuged again for 10 min. Blank samples did not contain either substrate or trapping agent. The duplicate samples were measured with an LC/MS spectrometer.

### 2.4 LC/MS analysis of the adducts

The peptides or GSH were adducted with the substrates in vitro in the presence of the enzyme source, and the fragment ions of adducts were analyzed by LC/MS. Each comparative experiment in this study was incubated and analyzed as a single batch. LC separation was carried out using an Agilent 1200 Series Binary Pump SL pump system and an Agilent 1200 Autosampler (Agilent Technologies, Inc., Santa Clara, CA, USA) equipped with an Agilent Zorbax SB-C18 Rapid Resolution HT column (150 mm × 2.1 mm, 1.8 μm) and a Phenomenex Gemini-NX C18 guard column (4 mm × 2.0 mm) (Phenomenex, Torrance, CA, USA). The injection volume was 10 μl and the flow rate was 100 μl/min. Eluent A was 0.1% formic acid in water and eluent B was 0.1% formic acid in 90% acetonitrile and 10% water. Eluent B was held at 5% for 2 min, then increased from 5% to 100% in 15 min and held at 100% B for 1 min. The gradient was then decreased from 100% to 5% B in 1 min and the column was stabilized by 11 min flow of 5% eluent B. Autosampler temperature was 10 °C and column temperature was held at 35 °C.

A Finnigan LTQ linear ion trap mass spectrometer was used for the detection of the formed adducts. The instrument was equipped with an electrospray ionization source and operated in the positive ion mode. A divert valve was used to direct the eluent flow to the mass spectrometer from 6 to 22 min of the LC run. The spray was stabilized with a nitrogen sheath flow with a value set to 30 instrument units, the spray needle voltage was 4 kV, the stainless steel capillary temperature was 230 °C and the capillary voltage was 19 V. The collision energy was 40 V and the isolation width used was 2.0 for MS2. Full-scan mass spectra were used to verify the molecular weights of the analysed adducts. The qualitative identification of the GSH and peptide adduct profiles formed was based on their typical MS2 fragment ions, which were predicted using ProteinProspector software (available at: http://prospector.ucsf.edu/prospector/cgi-bin/mssearch.cgi). Data acquisition was performed using Xcalibur DATA System 2.0 software (Thermo Electron Corporation, Waltham, MA, USA).

## 3 Results and discussion

Detection of reactive metabolites is based on their reactivity and conjugation with trapping agents. The synthesized d-peptides used in the study contained nucleophilic lysine, cysteine, histidine or arginine, which can react with electrophilic compounds. “Soft” electrophiles (quinones, quinone imines, epoxides, arene oxides and nitrenium ions) react primarily with “soft” nucleophiles such as the sulfhydryl group in cysteine. “Hard” electrophiles (such as aldehydes) react preferentially with “hard” nucleophiles such as lysine, histidine and arginine. We tested the qualitative trapping properties of three synthetic d-peptides with different sequences and compared their binding properties to that of GSH using mouse and human liver microsomes as catalyzing enzyme source. Adduct formation and fragment ion identifications were evaluated with six structurally different compounds: amlodipine, caffeine, clozapine, ibuprofen, nimesulide and ticlopidine (Table 1). The adducts formed were analyzed by LC/MS ion-trap mass-spectrometry with an ESI source. Species-dependent differences and similarities were observed in the formation of adducts: the “safe” compounds (amlodipine, caffeine, ibuprofen) resulted in divergent reading of adducts while the “risky” agents (clozapine, nimesulide, ticlopidine) produced adducts in both platforms and did not exhibit any “species-dependent” or trapping agent specificity in adduct formation. This consistent adduct reading for the “risky” compounds suggests unequivocally that reactive metabolite(s) can bind to any of the potentially reactive amino acid residues. However, different peptides exhibited their own distinctive trapping characteristics because of their different amino acid sequences, which are distinct from that of the gold standard GSH, which has the fixed sequence: *γ*-glu–cys–gly and the nucleophilic thiol of the cysteine residue.

**Table 1:** The trapping agents, the substrates, the formed adducts with their retention times and fragment ions. GSH and D-peptiDe adducts of test compounds. The substrates (50 μM) were incubated with mouse or human liver microsomes at CYP oxidizing conditions in the presence of GSH or peptide and then analysed by LC/MS ion-trap mass-spectrometry with an electrospray ionization source. -, no adduct or fragment ion could be identified.

GSH adduct					
	Substrate	Mouse, t_R_ (min)	Fragment ions	Human, t_R_ (min)	Fragment ions
Clinically safe	Amlodipine	–	–	–	–
	Caffeine	–	–	–	–
	Ibuprofen	–	–	–	–
Clinically risky	Clozapine	+GSH (14.3)	316.7(M + 2H, calc 316.6), 632.2 (MH, calc 632.2), 503.2 (y2, calc 503.2), 614.2 (M + H−H2O, calc 614.2), 359.2 (Drug + SH, calc 359.1)	+GSH (14.3)	316.6(M + 2H, calc 316.6), 632.3 (MH, calc 632.2), 503.1 (y2, calc 503.2), 614.1 (M + H−H2O, calc 614.2), 359.2 (Drug + SH, calc 359.1)
			316.6(M + 2H, calc 316.6), 503.2 (y2, calc 503.2), 359.2 (Drug + SH, calc 359.1)		316.6(M + 2H, calc 316.6), 503.2 (y2, calc 503.2), 359.2 (Drug + SH, calc 359.1)
			309.6 (M + 2H, calc 309.6), 618.1 (M + H, calc 618.2), 489.2 (y2, calc 489.1), 345.1 (Drug−CH2 + SH, calc 345.1)		–
		+GSH (14.1)	–	+GSH (14.1)	
					324.7 (M + 2H, calc 324.6), 648.2 (M + H, calc 648.2), 519.2 (y2, calc 519.2), 630.1 (M−H2O, calc 630.2)
		−CH_2_ + GSH (14.0)		–	
		–		+O + GSH (14.5)	
	Nimesulide	-NO_2_ + OH + GSH (14.9)	585.0 (M + H, calc 585.1), 456.1 (y2, calc 456.1)	-NO_2 _+ OH n+ GSH (14.9)	585.0 (M + H, calc 585.1), 456.1 (y2, calc 456.1)
			584.1 (M + H, calc 584.1), 455.1 (y2, calc 455.1)		584.1 (M + H, calc 584.1), 455.1 (y2, calc 455.1)
		−2O + 2H + GSH (15.0)		−2O + 2H + GSH (15.0)	
	Ticlopidine	+GSH (14.3)	569.0 (M + H, calc 569.1), 440.1 (y2, calc 440.1), 551.2 (M + H−H2O, calc 551.1)	–	
			569.0 (M + H, calc 569.1), 440.0 (y2, calc 440.1), 551.2 (M + H−H2O, calc 551.1)		
		+GSH (14.7)		–	
			294.2 (M + 2H, calc 294.1), 587.1 (M + H, calc 587.1), 458.1 (y2, calc 458.1), 569.2 (M + H−H2O, calc 569.1)		
		+2H+O+GSH (12.2)	294.2 (M + 2H, calc 294.1), 587.1 (M + H, calc 587.1), 458.1 (y2, calc 458.1), 569.2 (M + H−H2O, calc 569.1)	+2H+O+GSH (12.2)	294.2 (M + 2H, calc 294.1), 587.1 (M + H, calc 587.1), 458.1 (y2, calc 458.1), 569.1 (M + H−H2O, calc 569.1)
			302.2 (M + 2H, calc 302.1), 603.1 (M + H, calc 603.1), 474.1 (y2, calc 474.1), 585.1 (M + H−H2O, calc 585.1)		294.2 (M + 2H, calc 294.1), 587.1 (M + H, calc 587.1), 458.0 (y2, calc 458.1), 569.1 (M + H−H2O, calc 569.1)
		+2H + O + GSH (13.0)	571.0 (M + H, calc 571.1), 264.1 (Drug + H, calc 264.1), 308.1 (GSH + H, calc 308.1	+2H+O+GSH (13.0)	302.1 (M + 2H, calc 302.1), 603.1 (M + H, calc 603.1), 474.1 (y2, calc 474.1), 585.2 (M + H-H2O, calc 585.1)
			301.1 (M + 2H, calc 301.1), 601.1 (M + H, calc 601.1), 472.1 (y2, calc 472.1), 583.0 (M + H−H2O, calc 583.0)		
		+2H + 2O + GSH (13.3)		+2H + 2O + GSH (13.3)	
		+2H + GSH (13.4)		–	
		+2O + GSH (13.7)		–	

Peptide 1 (gly–tyr–pro–cys–pro–his–pro) adduct					
Clinically safe	Substrate	Mouse, tR (min)	Fragment ions	Human, tR (min)	Fragment ions
	Amlodipine	–		–	
	Caffeine	–			742.3 (y5, calc 742.3), 371.7 (y5 +2, calc 371.7), 847.0 (b6, calc 847.3), 193.1 (a2, calc 193.1), 221.1 (b2, calc 221.1)
	Ibuprofen	+O + pep (13.8)* –	770.2 (y5, calc 770.4), 385.6 (y5 +2, calc 385.7), 875.8 (b6, calc 875.4)	– +O + pep (14.4)	495.6 (M + 2H, calc 495.7), 350.2 (y3, calc 350.2), 673.3 (y4, 673.3), 770.3 (y5, calc 770.4), 385.7 (y5 +2, calc 385.7), 193.1 (a2, calc 193.1), 221.0 (b2, calc 221.0), 875.3 (b6, calc 875.4)

Clinically risky	Clozapine	+pep (14.0) –	547.7 (M+2H, calc 547.7), 874.2 (y5, calc 874.3), 437.8 (y5 +2, calc 437.7), 979.2 (b6, calc 979.4), 490.3 (b6 +2, calc 490.2), 359.1 (Drug + SH, calc 359.1)	+pep (14.0)	547.7 (M + 2H, calc 547.7), 874.2 (y5, calc 874.3), 437.8 (y5 +2, calc 437.7), 979.2 (b6, calc 979.4), 490.2 (b6 +2, calc 490.2), 359.2 (Drug + SH, calc 359.1)
	Nimesulide	-2O + 2H + pep (15.0)*	826.2 (y5, calc 826.3), 413.8 (y5 +2, calc 413.7), 221.0 (b2, calc 221.1), 931.1 (b6, calc 931.3)	−2O + 2H + pep (15.0)*	826.2 (y5, calc 826.3), 413.6 (y5 +2, calc 413.7), 221.0 (b2, calc 221.1)
	Ticlopidine	–	–	+2H + O + pep (13.9)	525.2 (M + 2H, calc 525.2), 829.1 (y5, calc 829.3), 415.2 (y5 +2, calc 415.2), 193.1 (a2, calc 193.1), 221.0 (b2, calc 221.0), 934.1 (b6, calc 934.3), 467.7 (b6 +2, calc 467.7)
		–	–	+2H + 2O + pep (14.1)*	845.2 (y5, calc 845.3), 423.1 (y5 +2, calc 423.1), 193.1 (a2, calc 193.1)
		–	–	+2H + 2O + pep (14.9)*	845.0 (y5, calc 845.3), 423.0 (y5 +2, calc 423.1), 193.1 (a2, calc 193.1), 221.1 (b2, calc 221.0), 950.0 (b6, calc 950.3), 296.1 (M + H−peptide calc 296.1), 770.2 (M + Hdrug, calc 770.3)
		–	–	+2H + pep (14.2)	517.1 (M + 2H, calc 517.2), 264.1 (M + Hpeptide, calc 264.1), 770.2 (M + H−drug, calc 770.3)
		–	–	+H−Cl + pep (14.5)	499.2 (M + 2H, calc 499.2), 648.1 (b4, calc 648.2), 745.1 (b5, 745.3), 882.1 (b6, calc 882.3), 441.7 (b6 +2, calc 441.7)

Peptide 2 (gly–tyr–pro–ala–pro–his–pro) adduct					
	Substrate	Mouse, t_R_ (min)	Fragment ions	Human, t_R_ (min)	Fragment ions
Clinically safe	Amlodipine	–	–	–	–
	Caffeine	–	–	–	–
	Ibuprofen	–	–	–	–
Clinically risky	Clozapine	–	–	–	–
	Nimesulide	–	–	–	–
	Ticlopidine	+pep(14.7)	500.2 (M + 2H, calc 500.2), 650.2 (b4, calc 650.2), 747.2 (b5, calc 747.3), 884.2 (b6, calc 884.3), 442.7 (b6 +2, calc 442.7), 428.6 (a6 +2, calc 428.7)	+pep(14.7)*	482.1 (b2, calc 482.1), 650.1 (b4, calc 650.2), 747.2 (b5, calc 747.3), 884.3 (b6, calc 884.3), 442.7 (b6 +2, calc 442.7), 428.7 (a6 +2, calc 428.7)
			500.2 (M + 2H, calc 500.2), 518.2 (y5, calc 518.3), 681.3 (y6, calc 681.3), 482.0 (b2, calc 482.1), 442.6 (b6 +2, calc 442.7), 262.0 (M+H-peptide, calc 262.1), 738.2 (M + H−drug, calc 738.4)		500.2 (M + 2H, calc 500.2), 518.2 (y5, calc 518.3) 482.1 (b2, calc 482.1), 262.1 (M + H-peptide, calc 262.1), 738.4 (M + H−drug, calc 738.4)
			516.1 (M + 2H, calc 516.2), 518.7 (y5, calc 518.3), 916.0 (b6, calc 916.3), 458.7 (b6 +2, calc 458.7), 294.0 (M + H−peptide, calc 294.1), 738.2 (M + H−drug, calc 738.4)		
		+pep (15.7)		+pep (15.7)	516.3 (M + 2H, calc 516.2), 916.1 (b6, calc 916.3), 458.7 (b6 +2, calc 458.7), 294.0 (M + H-peptide, calc 294.1), 738.2 (M + H−drug, calc 738.4)
			484.2 (M + 2H, calc 484.2), 518.3 (y5, calc 518.3), 618.3 (b4, calc 618.3), 715.3 (b5, calc 715.3), 852.1 (b6, calc 852.4), 426.7 (b6 +2, 426.7), 412.6 (a6 +2, calc 412.7), 435.8 (b6 + H2O +2, calc 435.7)		
		+2O + pep (15.7)		+2O + pep (15.7)	
		−S−2H + pep (14.6)		–	

Peptide 3 (gly–tyr–arg–pro–cys–pro–his–lys–pro) adduct					
	Substrate	Mouse, t_R_ (min) Fragment ions		Human, t_R_ (min) Fragment ions	
Clinically safe	Amlodipine	–	–	–	–
	Caffeine	–	–	–	–
	Ibuprofen	–	–	–	–
Clinically risky	Clozapine	+pep (12.6)	689.7 (M + 2H, calc 689.8), 1135.4 (b7, calc 1135.5), 568.3 (b7 +2, calc 568.2), 632.5 (b8 +2, calc 632.3)	+pep (12.6)	689.9 (M + 2H, calc 689.8), 1135.3 (b7, calc 1135.5), 568.2 (b7 +2, calc 568.2), 632.4 (b8 +2, calc 632.3)
	Nimesulide	–		-NO_2_ + OH + pep (12.8)	666.4 (M + 2H, calc 666.3), 1088.4 (b7, calc 1088.4), 544.6 (b7 +2, calc 544.7), 1216.9 (b8, calc 1216.5), 608.8 (b8 +2, calc 608.8)
	Ticlopidine	–	–	–	–

* Due to low signal, full scan mass spectrum could not be obtained.

Rationale behind the used abbreviations: +GSH/peptide, direct adduction with GSH/peptide; −CH2 + GSH/peptide, demethylation reaction before adduction with GSH; +O + GSH/peptide, introduction of O molecule and adduction with GSH/peptide; −NO2 + OH + GSH/peptide, elimination of NO2 group, introducing an OH group and adduction with GSH/peptide; −2O + 2H + GSH/peptide, two oxygen molecules removed, two H atoms added and adducted with GSH/peptide; +2O + GSH/peptide, two O molecules added and adducted with GSH/peptide; +2H + GSH/peptide, two H molecules removed and adducted with GSH/peptide; +H + peptide, two H molecules added and adducted with peptide.

Bioanalytical techniques, including the detection of GSH adducts and covalent binding assessment using radiolabeled chemical entities, have been used during lead optimization in the nonclinical phase of drug development to identify and minimize reactive metabolite formation [3], [5], [10]. The formation of reactive metabolites and their covalent binding to macromolecules are thought to be important — but not the only processes in the toxic reactions evoked by drugs.

Amlodipine has been considered as a very safe drug for hypertension [18]. It is one of the 1,4-dihydropyridine calcium channel blockers and it does not contain any obvious structural alerts. In this test, amlodipine did not produce any adducts with either GSH or any of the peptides, irrespective of whether the activating system was based on human or mouse liver microsomes. However, this drug is known to be metabolized to at least 21 different stable metabolites in primary rat hepatocytes [47]. The lack of GSH or peptide targeting adduct with a human enzyme source refers to the lack of generation of reactive intermediates in our test platform.

Caffeine (1,3,7-trimethylxanthine) is generally recognized as “safe” with moderate daily use, although it is subject to metabolism through several pathways. Caffeine can be oxidized at four positions: 3-*N*-demethylation to produce paraxanthine, 1-*N*-demethylation to produce theobromine, 7-*N*-demethylation to produce theophylline, and 8-hydroxylation to produce 1,3,7-trimethyluric acid. 3-*N*-demethylation is the main oxidation pathway of caffeine in human liver microsomes as compared to 1-*N*- and 7-*N*-demethylation and 3-*N*-demethylation [4]. Perhaps because of the several functionalization reactions that can occur, caffeine formed an adduct with Peptide1 in the incubation with human liver microsomes that has not been previously described in the literature. However, no adducts were found with GSH or peptide 2 and 3.

**Fig. 2:**
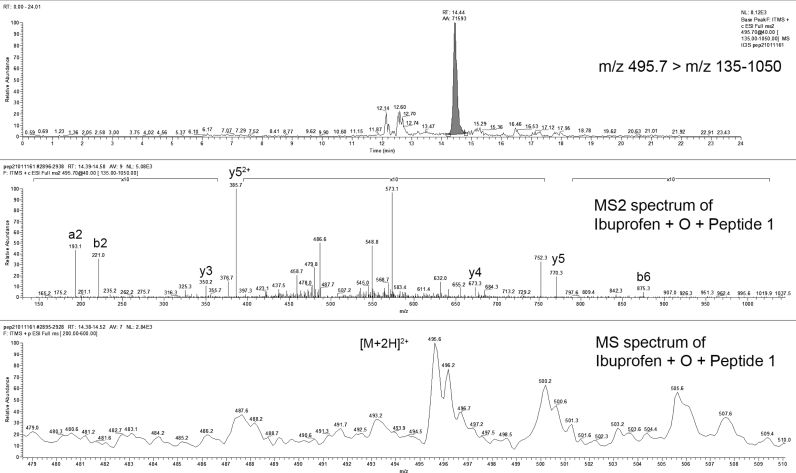
Mass spectrum of ibuprofen-Peptide 1 (gly–tyr–pro–cys–pro–his–pro) adduct.

Ibuprofen is a well-tolerated nonsteroidal anti-inflammatory drug (NSAID) causing only a low incidence of serious adverse reactions [46]. It is metabolized to form an acyl glucuronide, as well as carboxyibuprofen and hydroxyibuprofen, which are excreted in the urine together with the parent drug Geisslinger, 1989. Ibuprofen has been shown to react catalytically with several proteins including the UDP-glucuronosyltransferases, which catalyze these conjugation reactions [41]. In our study, ibuprofen formed only one adduct in incubations with both human and mouse liver microsomes (+ O + peptide) with Peptide 1 (Fig. 2; Table 1) and no other adducts were formed with any other trapping agents. However, these adducts had different retention times with mouse (13.8) and human (14.4) tissue. This may be due to tissue-specific differences in metabolism, such that the trapping agent is binding to different reactive sites on the ibuprofen molecule. Recently, a report appeared about novel metabolites of ibuprofen, namely ibuprofen-*N*-acyl-cysteinylglycine, ibuprofen-*N*-acyl-cysteine, and the mercapturic acid conjugate, ibuprofen-*S*-acyl-*N*-acetylcysteine [11]. Based on the clinical safety profile, the toxicological significance of the adduct is questionable, so that this may be an example of an adduction without toxicological significance. Once again, the lack of GSH, peptide 2 and 3 adducts with a human enzyme appears to be a reflection of the good clinical safety profile of the drug.

Clozapine is an antipsychotic drug with a well-known potential to cause adverse reactions in its clinical use, such as myocarditis or cardiomyopathy [12], [50] and fatal neutropenia, agranulocytosis, and hepatotoxicity [1], [30]. Clozapine is metabolized by cytochrome P450 (CYP) enzymes to form reactive metabolites such as a nitrenium ion, resulting in activated neutrophils and bone marrow cells [35]. In our tests, clozapine formed three adducts with GSH with human and mouse liver microsomes. Two clozapine + GSH adducts were identified; these compounds were slightly different with retention times 14.3 and 14.1 min. This is possibly due to the differences in the binding site of clozapine on the GSH molecule. The third adduct was different when incubated with the human and mouse biocatalysts. With mouse microsomes, the adduct –CH_2_ + GSH was formed with a retention time of 14.0 min and with human microsomes + O + GSH had a retention time of 14.5 min. Both of the cysteine containing peptides, 1 and 3, detected one adduct (+peptide) with both liver microsomes, but no adduct was detected with peptide 2 missing the cysteine residue. In line with our data, five clozapine-GSH conjugates have been detected [2], [10], [43], [37], [53], [60]. Additionally, covalent adducts of clozapine with proteins have been detected by Western blotting [55] and covalent protein modifications with electrochemistry/LC/MS [27]. Clozapine has been shown to modify rat liver proteins, forming covalent adducts with rat liver cytosolic and membrane fractions and a protein adduct in the bone marrow of rats [7]. In line with these data, it is proposed that clozapine–peptide adducts could serve as a surrogate endpoint to mimic protein adducting characteristics, in situations where the widely used trapping agent GSH will be overwhelmed. These results suggest that short peptides with a cysteine residue(s) could be designed to mimic potential target proteins, and used as a tool to identify/detect adverse responses caused by reactive metabolites.

Nimesulide is an anti-inflammatory drug that has caused hepatotoxicity [48]. Its use is known to deplete GSH stores and to evoke mitochondrial energy supply uncoupling and oxidative stress. Previously, several human nimesulide urinary metabolites were identified, including hydroxynimesulide, amino des-nitronimesulide, amino hydroxynimesulide and *N*-acetylated metabolites. Most of the metabolites are naturally stable and not electrophilic. Moreover, the idiosyncratic toxicity associated with nimesulide cannot be directly attributed to any of the known metabolites. However, these metabolites exhibit structural features that could enable them to be oxidatively bioactivated to reactive electrophilic species that can form adducts with macromolecules and peptides and may be capable of triggering a toxic response. The amino des-nitronimesulide metabolite is a highly electrophilic di-iminiquinone and is capable of forming adducts with *N*-acetylcysteine [24]. In the present study, nimesulide formed two adducts (−NO_2_ + OH + GSH and −2O + 2H + GSH) with GSH when incubated with both microsomes. With Peptide 1, one adduct (−2O + 2H + peptide) was formed with both microsomes. Peptide 3 formed one adduct with human liver microsomes (−NO_2_ + OH + peptide). These data confirm that nimesulide possesses macromolecule-adducting characteristics. Once again, the consistent formation of GSH adducts with both species and peptide adducts to cysteine residue containing peptides support the hazardous nature of nimesulide.

Ticlopidine is a drug used to inhibit the actions of thrombocytes. It can cause serious hematological disorders [15] and liver toxicity Previteral and Pagani, 2010. The thiophene moiety of ticlopidine is mainly oxidized by CYP2C19 in humans, leading to the formation of electrophilic reactive metabolites, including thiophene S-oxide and thiophene epoxide, which can undergo covalent binding to cellular proteins including CYP2C19 [13]. In our study, ticlopidine formed seven adducts with GSH (+GSH (2), +2H + O + GSH (2), +2H + 2O + GSH, +2H + GSH, +2O + GSH) with mouse liver microsomes. Of these adducts, two slightly different + GSH adducts (with retention times 14.3 and 14.7 min) were identified and two similarly different adducts +2H + O + GSH (with retention times 12.2 and 13.0 min). That is possibly due to stereochemistry and differences in the binding site of ticlopidine on the GSH molecule. Two GSH adducts (+2H + O + GSH and +2H + 2O + GSH) were also found with human liver microsomes, of which +2H + O + GSH had two forms (with retention times 14.3 and 14.7 min). With Peptide 1, five adducts were formed with human liver microsomes (+2H + O + peptide (2), +2H + 2O + peptide, +2H + peptide, +H −Cl + peptide), but none with mouse liver microsomes. With respect to the adducts formed after incubation with human microsomes, two different +2H + 2O + peptide adduct forms were found with retention times 14.1 and 14.9 min. With Peptide 2, four adducts were formed with mouse liver microsomes (+peptide (2), +2O + peptide and –S −2H + peptide). The + peptide adduct was present in two forms with retention times 14.7 and 15.7 min. With human liver microsomes, three adducts (+peptide (2) and 2O + peptide) were identified, and similarly the + peptide adduct existed in two different forms (retention times 14.7 and 15.7 min). According to the literature, ticlopidine has been shown to form different types of adducts with GSH [37], (+GSH), (+O + GSH), (+2H + O + GSH) and (+2H + 2O + GSH) [2], [53]. Direct conjugation of ticlopidine to GSH has also been reported by [26], i.e., the site of conjugation is on one side of the molecule, namely the thiophene ring. It should be noted that the presence of the thiophene moiety has been associated with a significant incidence of adverse reactions in other drugs [28]. The wider number of ticlopidine-GSH adducts and the adduction with two d-peptides reveal the overall reactivity of this compound.

Based on the above data from six different compounds, it is possible to use short synthetic peptides for hazard identification and to develop them further to identify reactive metabolites. In some cases, this can give additional information of reactive metabolite formation compared to GSH. For instance, could a peptide adduct profile predict hapten-based immunological reactivity? Based on our data, GSH adduction as such, should still be considered as the gold standard for predicting the bioreactivity of the compound and reactivity/toxicity is proportional to the diversity of different adducts detected. With “safe” compounds, no GSH adducts and only a few peptide adducts were found: with amlodipine no adducts were detected with either GSH or peptides. Caffeine formed one peptide adduct with Peptide 1 that contains a cysteine residue in the middle of the amino acid chain when human liver microsomes were used as a biocatalyst. Ibuprofen resulted in one Peptide 1 adduct with both microsomes. With the “risky” agents (clozapine, nimesulide and ticlopidine) the list of adducts identified was largely peptide-dependent; several adducts were formed with GSH as well as with the peptides. Species differences were observed in formation of adducts between human and mouse with the trapping peptides and GSH. This is obviously due to species-dependent differences in the metabolic characteristics of metabolizing enzymes [34].

Our d-peptide — based trapping method allows straightforward detection of the reactive metabolites by LC/MS based on fragment ion analysis. It is well known that d-peptides are resistant to certain proteases [6], [32], which can increase their half-life in analytes [39], [21]. In our case, the benefits are that the d-isomer peptides are not hydrolyzed during the incubations with microsomes, incubation times can be increased, and the relative amount of the formed adducts can be more reliably compared to each other. Our results also indicate convincingly that the sequence of peptide – existence of nucleophilic thiol of cysteine residue – affects adduction, which may be used to classify differently reacting active metabolites.

## 4 Conclusions

Detection of reactive metabolites is based on their conjugation with trapping agents simultaneously during their formation. In vitro conditions were established to test adduct formation of oxidized xenobiotics between three d-peptide isomers and GSH. Qualitative adduct formation was tested with six structurally different compounds: amlodipine, caffeine, clozapine, ibuprofen, nimesulide and ticlopidine. The adducts formed were isolated and identified based on their fragment ions in LC/MS. Clozapine, nimesulide and ticlopidine produced adducts with both enzymatic biocatalyst and trapping platforms (GSH or d-peptide), confirming the generation of reactive intermediates with toxic potential. The number of adducts was compound and d-peptide dependent. With “safe compounds”, no or at the most only a few adducts were formed and identified. Toxicological significance of the above mentioned adducts remains to be evaluated further together with other safety variables. The peptide trapping method can be further developed for the screening of reactive metabolites; the peptides can be designed so that they can represent the proteins likely to undergo adduct formation in the human body. From the clinical point of view, whether peptide-targeting adduction contributes to the idiosyncratic, allergic or other immunologically related adverse reactions as demonstrated by the “risky” compounds, remain to be resolved.
